# Genome-wide analysis of day/night DNA methylation differences in *Populus nigra*

**DOI:** 10.1371/journal.pone.0190299

**Published:** 2018-01-02

**Authors:** Chang-Jun Ding, Li-Xiong Liang, Shu Diao, Xiao-Hua Su, Bing-Yu Zhang

**Affiliations:** 1 State Key Laboratory of Tree Genetics and Breeding, Research Institute of Forestry, Chinese Academy of Forestry, Beijing, China; 2 Key Laboratory of Tree Breeding and Cultivation of State Forestry Administration, Research Institute of Forestry, Chinese Academy of Forestry, Beijing, China; University of Perugia, ITALY

## Abstract

DNA methylation is an important mechanism of epigenetic modification. Methylation changes during stress responses and developmental processes have been well studied; however, their role in plant adaptation to the day/night cycle is poorly understood. In this study, we detected global methylation patterns in leaves of the black poplar *Populus nigra* ‘N46’ at 8:00 and 24:00 by methylated DNA immunoprecipitation sequencing (MeDIP-seq). We found 10,027 and 10,242 genes to be methylated in the 8:00 and 24:00 samples, respectively. The methylated genes appeared to be involved in multiple biological processes, molecular functions, and cellular components, suggesting important roles for DNA methylation in poplar cells. Comparing the 8:00 and 24:00 samples, only 440 differentially methylated regions (DMRs) overlapped with genic regions, including 193 hyper- and 247 hypo-methylated DMRs, and may influence the expression of 137 downstream genes. Most hyper-methylated genes were associated with transferase activity, kinase activity, and phosphotransferase activity, whereas most hypo-methylated genes were associated with protein binding, ATP binding, and adenyl ribonucleotide binding, suggesting that different biological processes were activated during the day and night. Our results indicated that methylated genes were prevalent in the poplar genome, but that only a few of these participated in diurnal gene expression regulation.

## Introduction

DNA methylation is a mechanism of epigenetic modification in eukaryotic organisms. In plants, DNA methylation generally occurs at cytosine bases in three different sequence contexts: CG, CHG and CHH contexts (H = A, T, or C) [1,2]. The mechanisms responsible for the establishment and maintenance of cytosine methylation have been best studied in *Arabidopsis* [3], where *de novo* methylation is catalyzed by Domains-Rearranged Methyltransferases2 (DRM2), and the maintenance of DNA methylation can be classified into two types: CG and CHG methylation are catalyzed by DNA Methyltransferase1 (MET1) and Chromomethylase3 (CMT3), respectively; CHH methylation is methylated by DRM2 [2,3](Chan et al., 2005; Law and Jacobsen, 2010).

In both animals and plants, DNA methylation status might have effects on gene expression, splicing, and polyadenylation [4–6], and can influence DNA repair, recombination and meiotic crossover in euchromatic regions [7–9]. In plants, studies have indicated that DNA methylation plays an important role in parental imprinting, floral symmetry, flowering time, pigmentation, fruit ripening, sex determination, and stomatal development [10–17]. Therefore, variation in DNA-methylation may be functional and result in phenotypic variability in plants.

In recent years, increasing evidence has shown that DNA methylation is sensitive to extrinsic signals. For example, in the dandelion (*Taraxacum officinale*), alternative phenotypes may be induced by environmental stress due to DNA methylation changes [18]. Therefore, external environmental factors might influence gene expression through DNA methylation. Light can alter the promoter DNA methylation level in the suprachiasmatic nucleus (SCN) of the hypothalamus and reprogram circadian behavior in mice [19]. In plants, cold stress induced hypomethylation in *Antirrhinum* and growth temperature has been shown to positively correlate with the DNA methylation degree of Tam3 transposon [20]. In *Arabidopsis* elevated temperature have been shown to influence the expression of gene At3g50770 by DNA methylation changes in promoter region [21]. Additionally, different methylation levels were observed between plants grown in light and dark [22]. In the natural environment, plants are growing under constantly changing environmental conditions (light intensity, temperature and humidity etc.) over the course of a day, daily DNA methylation change are therefore expected in plant genomes.

Poplars (*Populus* L.) are model species in tree biology, and typically produce genetically identical clones quickly. Therefore, poplars are ideal plant materials for the study of DNA methylation changes under different environmental conditions. In a previous study, we didn’t detect consistent DNA methylation variations over the course of a day in mature leaves of the *P*. *nigra* clone ‘N46’ by methylation-sensitive amplification polymorphism method (MSAP) [23]. The MSAP can only detect the methylation changes of the CCGG sites, but not the CHG and CHH sites which are prevalent in plant genome. In this study, using the same clone, we detected changes in DNA methylation of the whole genome between day and night using the methylated-DNA immune precipitation sequencing (MeDIP-Seq) method. We found that methylated genes were prevalent in the poplar genome, but that only a few of these participated in diurnal gene expression regulation. We will discuss the prevalence of DNA methylation in the poplar genome and its participation in diurnal regulation.

## Materials and methods

### Ethics statement

The plants used in this study were propagated under permission from the state forestry administration of China. The location is not privately-owned.

### Plant materials and growth conditions

We used the *P*. *nigra* clone ‘N46’ in this experiment. The production of ‘N46’remets and growth conditions were as previously described [23]. After four months of culture in the greenhouse, four homogeneous plants selected and the 4^th^–6^th^ leaves from the top were collected from each plant at 8:00 and 24:00, yielding two plants for each time point. Based on the photoperiod at the sample collection day (5:19~19:20), samples collected at 8:00 and 24:00 were used to present the day and night sample respectively. All leaves were immediately frozen in liquid nitrogen and stored at –80°C.

### Genomic DNA extraction and MeDIP-Seq

Genomic DNA was isolated from mature leaves of *P*. *nigra* clone ‘N46’ using the DNeasy Plant Mini Kit (Qiagen), and purified according to the manufacturer’s instructions.

The MeDIP-seq DNA libraries were constructed according to the instruction manual for the MagMeDIP kit (Diagenode). Sonication was used to fragment genomic DNA to the size of 100–600 bp. A 3' single-base dA overhang on the end of each DNA fragment was ligated and then Illumina sequencing adapters were added to the ends by the Paired-End DNA Sample Prep Kit (Illumina). Denatured double-stranded DNA fragments were immunoprecipitated with 5-methylcytosine antibody (Diagenode). The quality of immunoprecipitated fragments were validated by quantitative real-time polymerase chain reaction (qPCR). DNA fragments of 200–300 bp were gel-excised and purified using the Qiagen gel extraction kit. The Quant-iT dsDNA High Sensitivity Assay Kit (Invitrogen) was used to quantify the extracted fragments on an Agilent 2100 Analyzer (Agilent Technologies, Germany). Then, DNA libraries were paired-end sequenced using the Illumina HiSeq 2000 platform (Illumina). The raw image files produced were analyzed by Illumina Real-Time Analysis (RTA) and base calling.

The adapters and low-quality reads were filtered out by Fastx (version: 0.0.13). The clean reads obtained were then aligned to the *Populus trichocarpa* reference genome (JGI 2.0.15). Genome mapping was performed using the Bowtie software (version: 0.12.8) in local alignment mode with default parameters to generate the BAM files.

### Identification of differentially methylated regions (DMRs)

Methylation-enriched regions (or peaks) were detected from the BAM files using ChIP-seq (MACS, version 1.4), and peaks with a *p*-value less than 1 × 10^−5^ were defined as passed peaks. The passed peaks were retained for further analyses.

The methylated exon, intron, promoter, coding sequence (CDS), 5′-untranslated region (UTR), 3′ UTR, and intergenic region (overlapped regions with DNA methylation peaks) were identified based on poplar genome database transcript annotations.

DMRs were identified using the method developed by Wang et al. [24]. If the peak detected in one sample overlapped with peaks in another sample, the genomic region covering these was selected as a candidate DMR region. The log_2_ ratio of 24:00 read numbers vs. those for 8:00 was calculated for each candidate DMR region, normalized, test *p*-value and using the DEGseq R package [25]. When *p* < 0.001 for the difference between the read numbers of each methylated region for two samples, we determined that the result was significant. The normalized log_2_ value and *p*-value were combined to determine whether a region was differentially methylated. DMRs with a greater than twofold difference in read numbers were classified as hypo- or hyper-methylated regions.

### Bisulfite sequencing of differentially methylated genes

To validate the MeDIP-Seq results, DMRs overlapping in five poplar genes were confirmed using bisulfite PCR (BS-PCR) and Sanger sequencing. Bisulfite conversions were completed using the DNA Bisulfite Conversion Kit (Tiangen). Bisulfite-modified PCR primers were designed using the online program BiSearch (http://bisearch.enzim.hu/?m=search). Information on the amplified methylated regions and primers are listed in Table 1. PCR was performed using GoTaq Hot Start Polymerase (Promega) in 20-μl reaction volumes containing 100 ng template DNA and 10 ng of primers. The PCR amplification conditions were: 7 min at 95°C, followed by 40 cycles of 40 s at 95°C, 40 s at 62°C and 1 min at 72°C, and further elongation at 72°C for 5 min using an ABI Veriti 96-Well Thermal Cycler (Applied Biosystems). The PCR products were gel separated, purified with an AxyPrep DNA gel extraction kit (Axygen). Purified amplicons were cloned into the pGEM-T Easy vector (Promega) and sequenced. The Sanger sequencing data were analyzed using MethTools 2.0 (http://194.167.139.26/methtools/MethTools2_submit.html) and the methylation rate was calculated by dividing the number of non-converted (methylated) cytosines by the total number of cytosines in the amplified fragment.

**Table 1:** Primers used in BS-PCR.

Genes ID	Amplification Length	Primer sequence	Genic Region
Potri.001G189900	399 bp	5’-CACGACAACTTTTCTAATGGAC-3’	Promoter
		5’-TGTCAGTGATGGTAAGGGACT-3’	
Potri.003G099100	450 bp	5’-GCTCAACGGAGGTAAAGCAC-3’	Promoter
		5’-GTGTTTTGGGTTGATTGATG-3’	
Potri.012G138800	376 bp	5’-GAGCTGATTGAGAGTTTAGT -3’	Promoter, CDS
		5’-CCATGTGAGGAAAAACTGTATC-3’	
Potri.007G044000	535 bp	5’-CGAATCTCATCATCCCGATT-3’	Promoter
		5’-CTATACCTTGCGGCCATGTT-3’	
Potri.002G180100	583 bp	5’-GGTCGAGAGACCGGATTTTA-3’	Promoter
		5’-GGGTCATTTTTGTGTTTTAGCC-3’	

### RNA isolation and qPCR analyses

Total RNA was extracted from mature leaves using RNeasy Plant Mini Kit (Qiagen) for gene expression validation experiments. After the treatment of RNase-Free DNase (Qiagen), each RNA sample was quantified using NanoDrop ND-1000. The cDNA was synthesized from 2 ug total RNA using moloney murine leukemia virus reverse transcriptase with an RNase inhibitor (Promega), at an annealing temperature of 37°C for 1 hour, then maintained at 65°C for 10 min to terminate the reaction.

We performed qPCR analysis in the LightCycler 480 System platform (Roche, Switzerland). Each 20 μl PCR system contained 1 μl of first-strand cDNA, 200 nM of primers and 1× SYBR PCR mixture (TaKaRa, Japan). The amplification conditions were: 10 s at 95°C, followed by 45 cycles of 10 s at 95°C, 10 s at 60°C and 10 s at 72°C. We performed three replicates for each sample. Relative quantification values were calculated using the 2^–⊿⊿CT^ method [26]. Amplification lengths and primers are listed in Table 2.

**Table 2:** Primers used in the qPCR.

Genes ID	Amplification Length	Primer sequence
Potri.012G138800	167 bp	5’-TGTGGATTGCTGCGATATTT-3’
		5’-AAAACCATGCGGAGAAACAC-3’
Potri.007G044000	157 bp	5’-TCCGTTCCACAAACAGATGA-3’
		5’-CTATACCTTGCGGCCATGTT-3’
Potri.002G180100	162 bp	5’-GACGATGCCACTCAGGTAGG-3’
		5’-CACACATGCATGCAAAATGA-3’
Potri.001G189900	192 bp	5’-AAACAGAAAGCAGAGGTTTCG-3’
		5’-CACGTCACACCTGATCTTGC-3’
Potri.003G099100	174 bp	5’-GCTCAACGGAGGTAAAGCAC-3’
		5’-CAAGTGCAGCAAAAGAGCTG-3’

## Results

### Day/night methylation profiles in *P*. *nigra* leaves

The MeDIP-seq data were generated from samples collected at 8:00 and 24:00. Following the removal of low-quality data, we obtained 37,039,173 reads (average length 100 bp) from the 8:00 samples and 35,953,618 reads from the 24:00 samples for further analysis. Approximately 48% of the MeDIP-seq reads could be mapped onto the poplar genome. The mapping rate in our study was similar to that in *P*.*trichocarpa* leaves (41.41%) [27], but much lower than that in rice (~85%), maize (~93%) and cotton (~95%) [24,28,29].

We detected a total of 74,565 methylation peaks from the 8:00 samples and 79,586 methylation peaks from the 24:00 samples (Table 3). More DNA methylation peaks indicate that more loci in the genome methylated [24]. These peaks were distributed across all 19 chromosomes, and the number of peaks in each chromosome was correlated with its length, indicating the prevalence of methylation in the poplar genome. Comparing the number of peaks in the samples for each time point, we found a slight increase in methylation level in the 24:00 samples.

**Table 3:** Statistics of the methylation peaks identified in 8:00 and 24:00 samples.

Sample	Total peaks	Mean length(bp)	Median length(bp)	Total length(bp)	Coverage (%)
8:00	74565	391.70	292	29,207,382	6.91
24:00	79586	361.23	437	28,749,163	6.80

**Fig 1:**
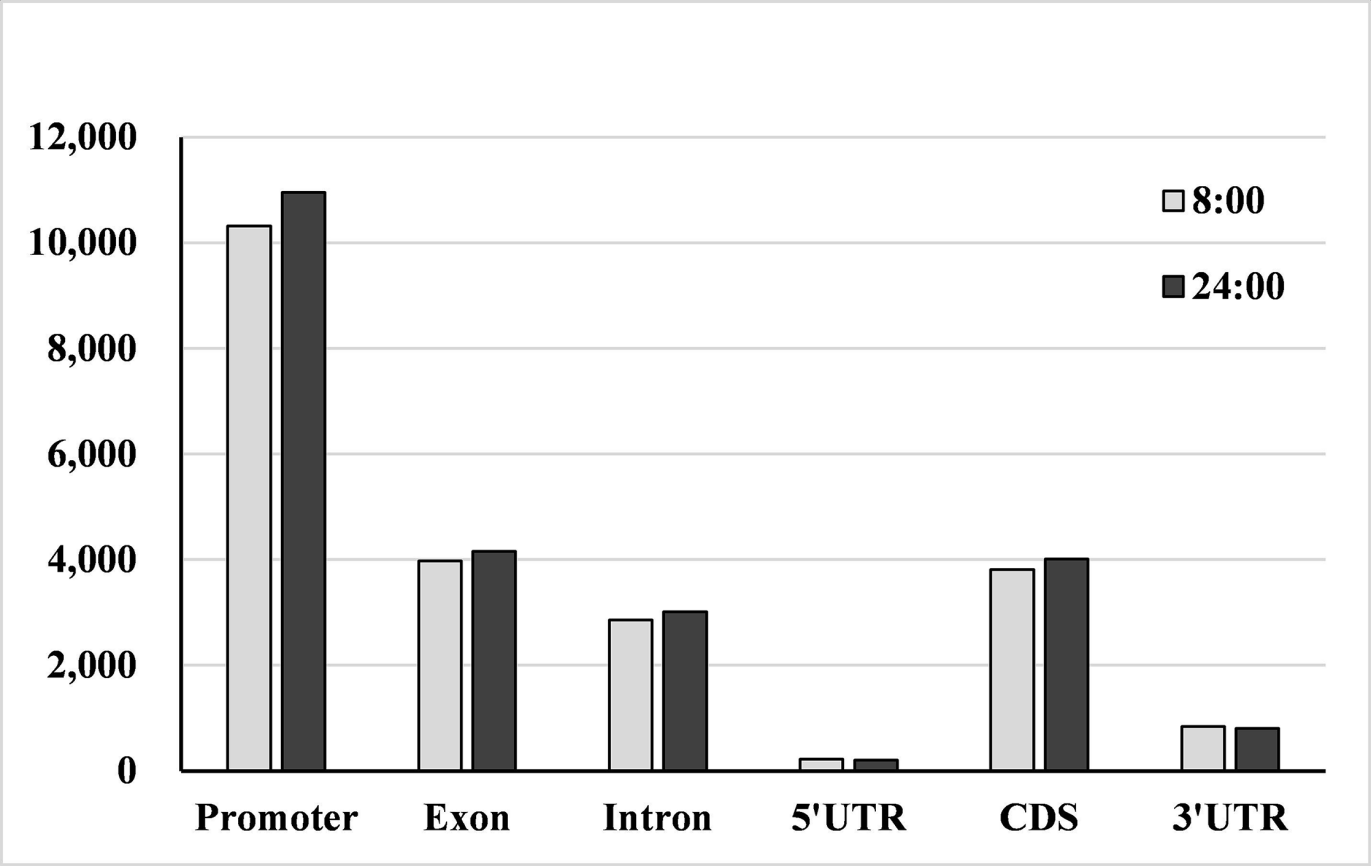
Distribution of methylation peaks in different genic regions of *P*.*nigra* genome. The methylation peaks were most often located in the promoters in both 8:00 and 24:00 samples.

**Fig 2:**
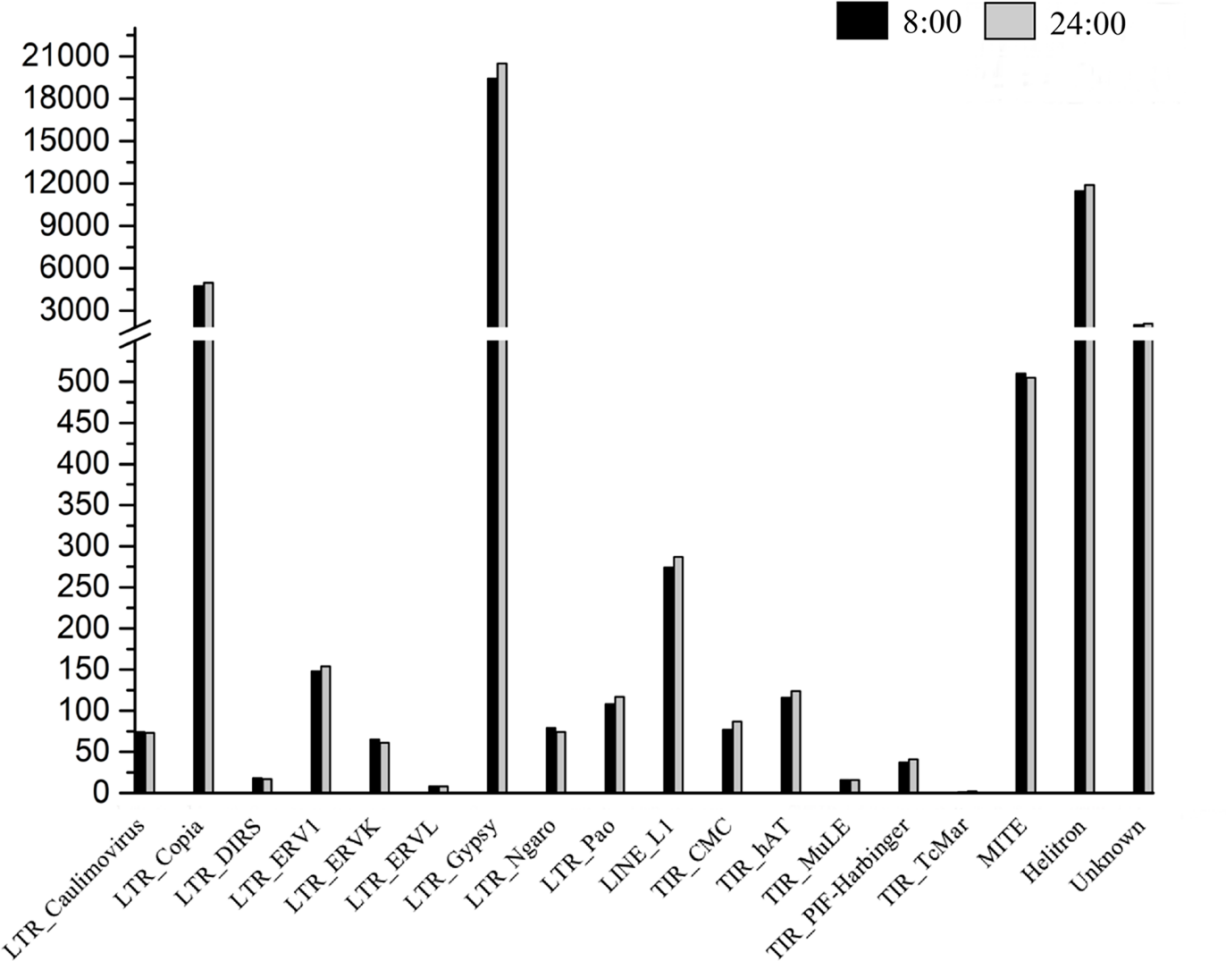
Distribution of methylation peaks in different TEs of *P*.*nigra* genome. The methylation peaks were seen in all the TE types, and the LTR_Gypsy, LTR_Copia and Hilitron tended to have much more methylated peaks than other TEs.

Genomic regions that overlapped with DNA methylation peaks were considered methylated [24]. Differential distribution of the identified peaks among genomic components was observed, with most methylation peaks located in the intergenic regions (approximately 81.5%). Within the genic regions, the methylation peaks were most often located in the promoters (47.06%) (Fig 1). The methylation peak distribution patterns of *P*. *nigra* in genic regions were similar to those of *Arabidopsis thaliana* [30] and *P*. *trichocarpa* [27]. Based on Transposable Elements Database of *P*.*trichocarpa* [31], distribution of peaks in TEs regions was analyzed. The methylation peaks were seen in all the TE types, and the LTR_Gypsy, LTR_Copia and Hilitron tended to have much more methylated peaks than other TEs, suggesting that the activity control of these types of transposons may be more important in poplars (Fig 2).

**Fig 3:**
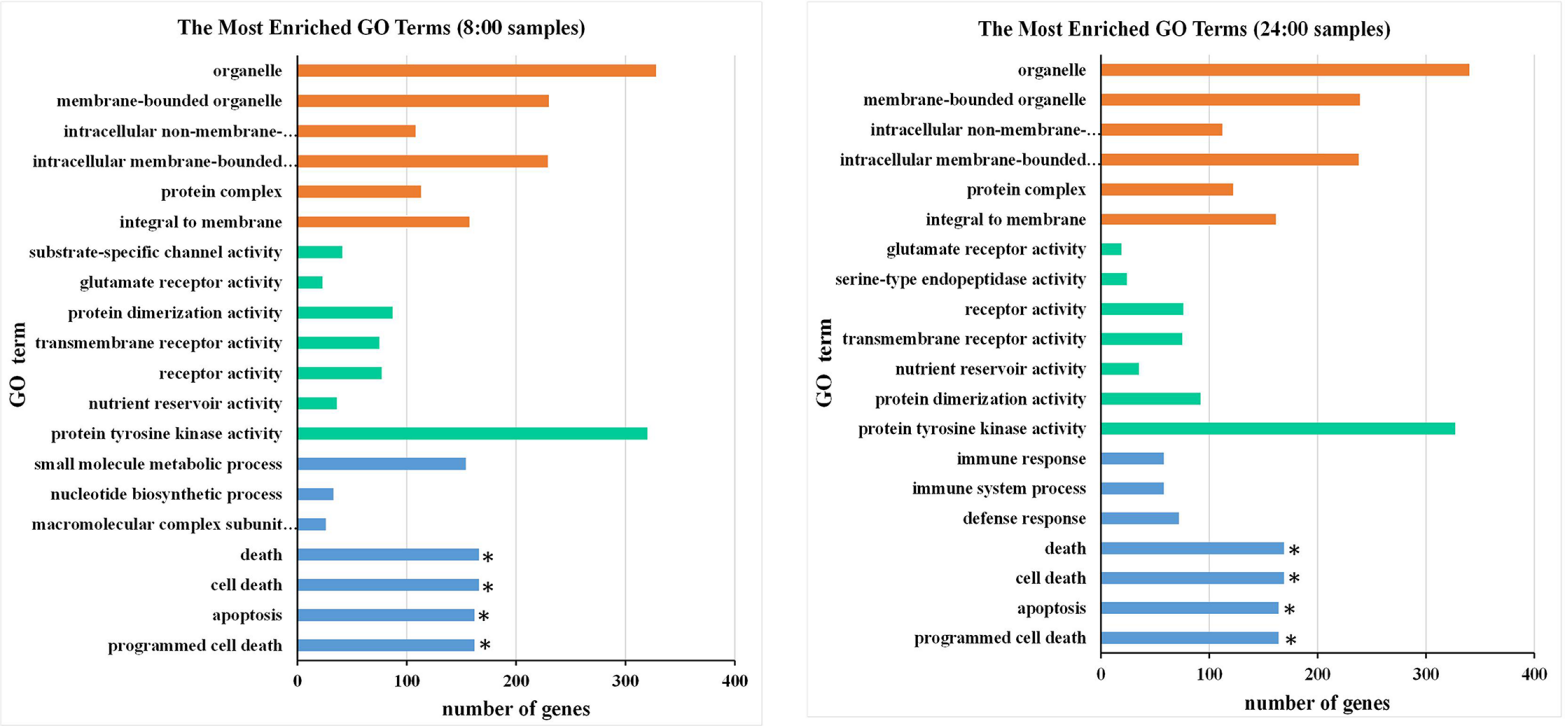
Significant enriched GO terms of the methylated genes in 8:00 and 24:00 samples. Significantly enriched methylated genes (corrected *p* value < 0.05, FDR < 0.05) appeared to be involved in death, cell death, programmed cell death, apoptosis, and protein binding.

Based on poplar genome database transcript annotation and the UniProtKB/TrEMBL database, the methylation peaks in the genic regions were function-annotated using the BLASTX program. The encoding protein of the methylated genes was further compared with proteins in the Kyoto Encyclopedia of Genes and Genomes (KEGG). In total, 10,027 and 10,242 methylated genes were annotated in the 8:00 and 24:00 samples, respectively. Gene ontology (GO) analysis of these methylated genes was performed by agriGO to detect the biological nature of the DNA methylation in the poplar genome. The methylated genes were annotated with up to 2,351and 2,443 GO terms by agriGO (http://bioinfo.cau.edu.cn/agriGO/), with 30.66% biological process terms, 21.02% cellular component terms, and 48.32% molecular function terms. The most enriched GO terms in the 8:00 and 24:00 samples were similar, such as protein phosphorylation and metabolic processes in the biological process GO domain; membrane, plasma membrane, chloroplast, and nucleus in the cellular component GO domain; and protein binding, ATP binding, nucleic acid binding, zinc ion binding, nucleotide binding, protein serine/threonine kinase activity, protein kinase activity, and catalytic activity in the molecular function GO domain. GO term enrichment analysis showed that significantly enriched methylated genes (corrected *p* value < 0.05, FDR < 0.05) appeared to be involved in death, cell death, programmed cell death, apoptosis, and protein binding (Fig 3). KEGG analysis showed that these methylated genes were involved in 128–129 pathways, including metabolic pathways, biosynthesis of secondary metabolites, plant hormone signal transduction, penylpropanoid biosynthesis, plant–pathogen interactions, carbon metabolism, and fatty acid metabolism. These results indicate that DNA methylation was prevalent in the poplar genome and may influence the expression of thousands of genes involved in a wide range of biological processes.

### Methylation changed between day and night in *P*. *nigra*

**Fig 4:**
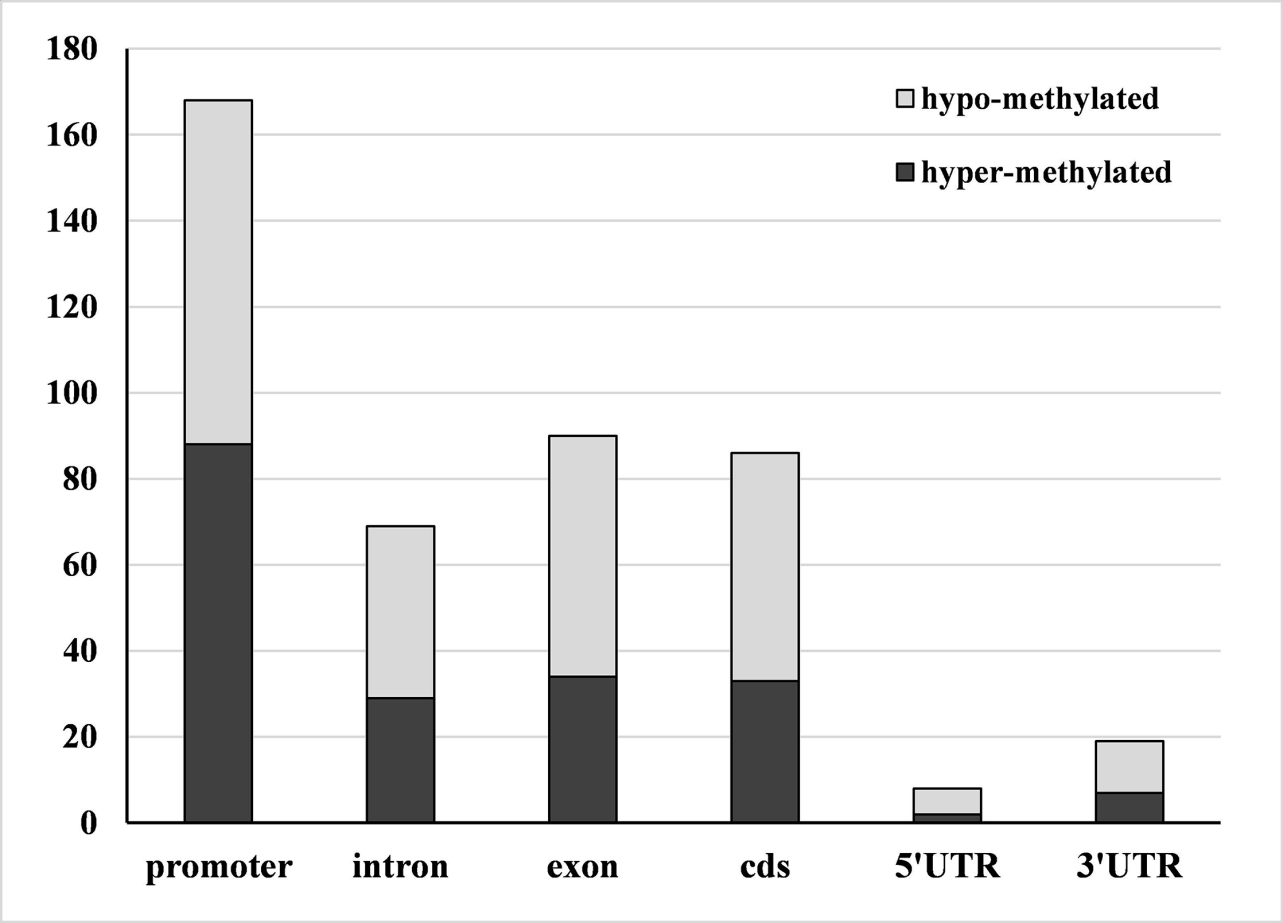
Distribution of DMRs in genic regions of 24:00 samples vs. 8:00 samples. Similar to the genic distribution of methylation peaks, the DMRs were most often located in the promoters in both 8:00 and 24:00 samples.

**Fig 5:**
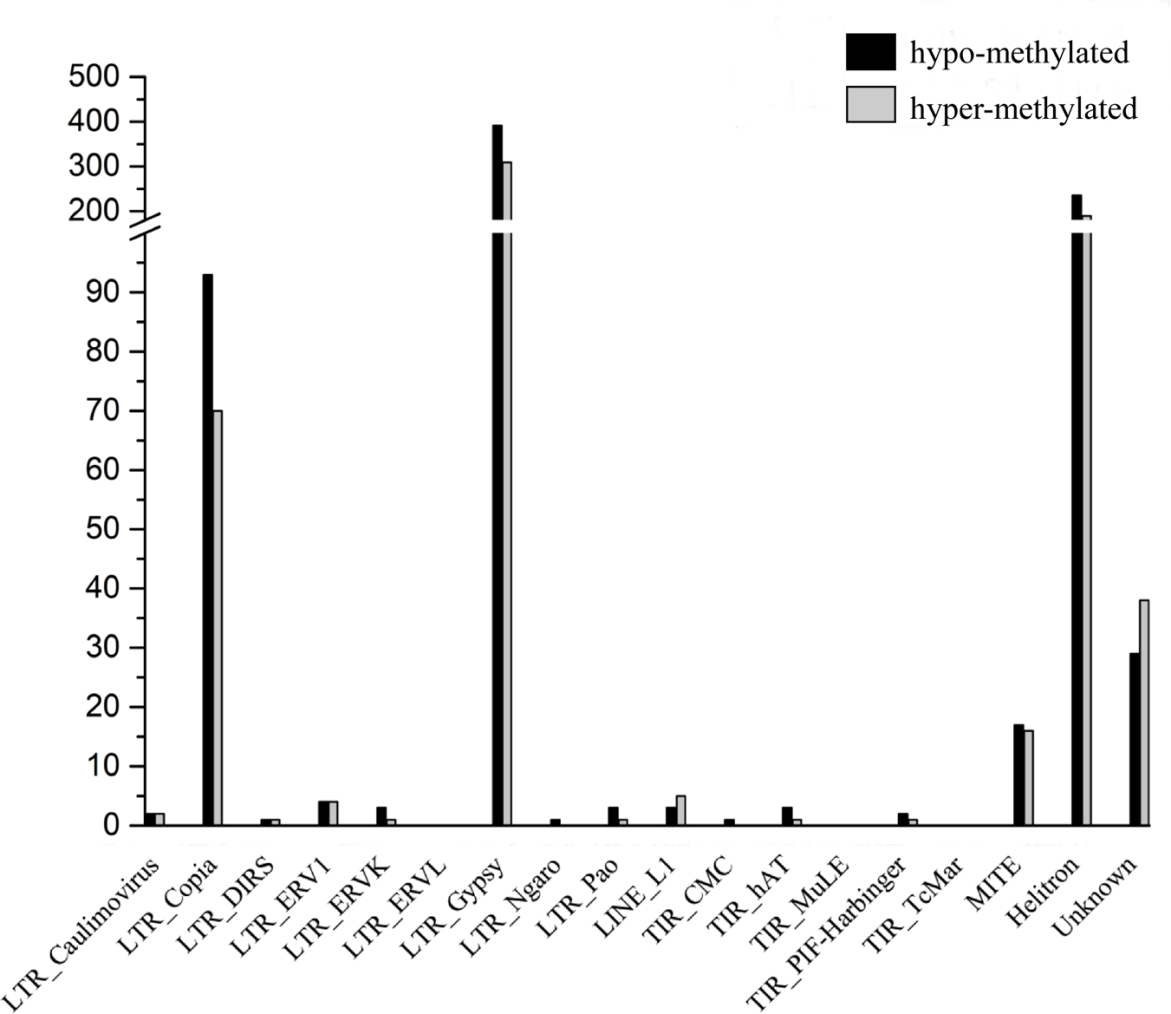
Distribution of DMRs in different TEs of *P*.*nigra* genome. Most DMRs located in the LTR_Gypsy, LTR_Copia and Hilitron transposons and no DMRs were seen in high levels of methylation were seen in LTR_ERVL, TIR_MuLE and TIR_TcMar.

In the present study, a total of 2,120 DMRs were identified between the 24:00 and 8:00 samples. Among the identified 2,120 DMRs, 952 and 1,168 regions were classified as hyper-methylated and hypo-methylated, respectively. To better describe the corresponding biological function of these DMRs, we identified DMRs that overlapped with gene regions; 193 hyper-methylated DMRs and 247 hypo-methylated DMRs were obtained. The distribution of DMRs in genic regions was similar to that of the peaks (Fig 4). For TEs regions, about 90.27% DMRs located in the LTR_Gypsy, LTR_Copia and Hilitron transposons and no DMRs were seen in high levels of methylation were seen in LTR_ERVL, TIR_MuLE and TIR_TcMar (Fig 5).

**Fig 6:**
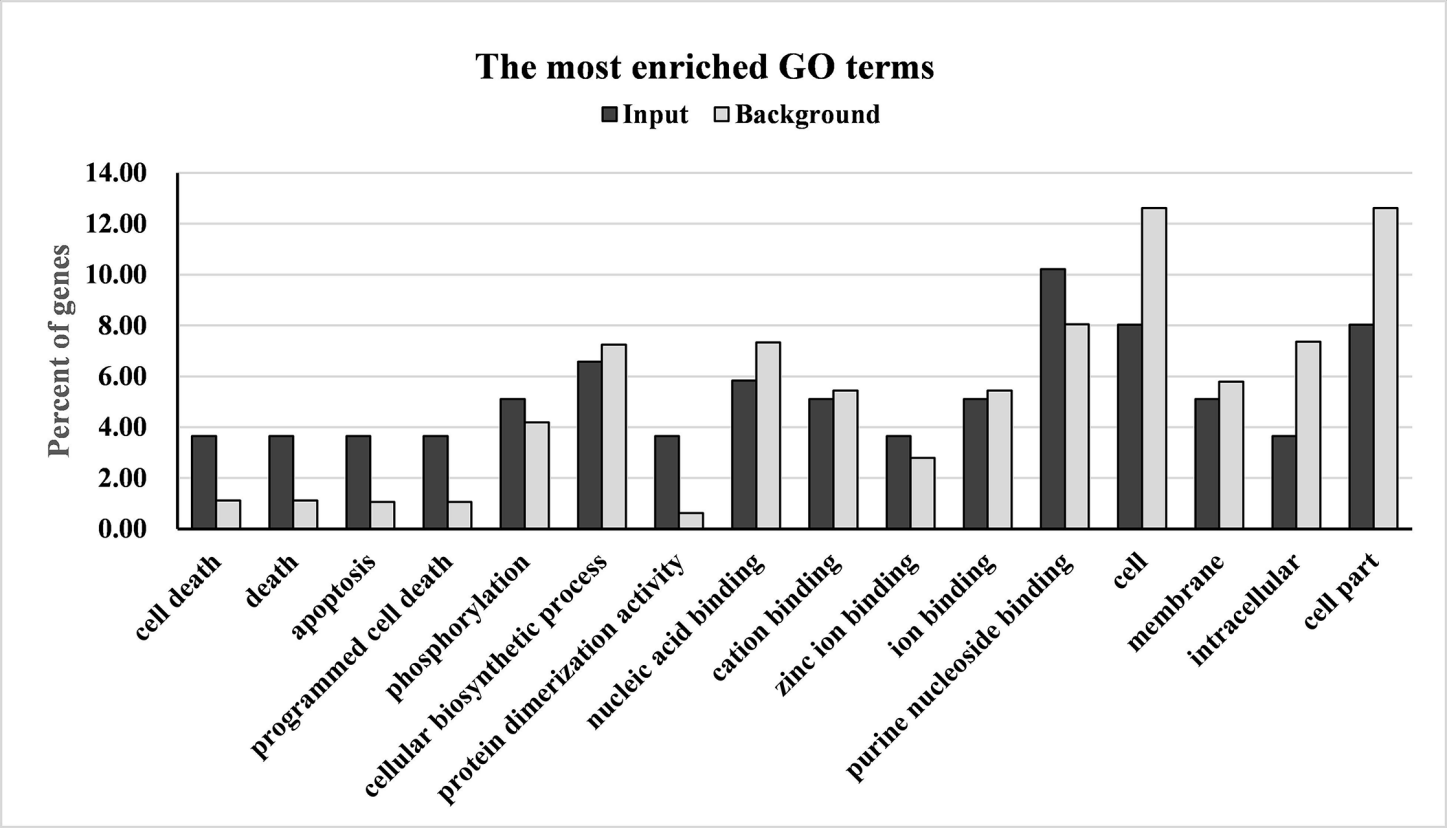
The most enriched GO terms of the DMR-overlapped genes in biological process, molecular function the cellular component.

In total, 137 genes overlapped with DMRs. The DMR-overlapped genes were annotated and subjected to GO analysis to detect the biological nature of the DNA methylation changes between day and night in the poplar genome. The DMR-overlapped genes were annotated to 56 GO terms by agriGO, with 4 in the cellular component, 26 in molecular function, and 26 in biological processes. No significantly enriched GO terms were found. The most enriched GO term in the cell component was the membrane; in molecular function the most enriched terms were transferase activity, kinase activity, phosphotransferase activity, ATP binding, protein binding, and adenyl ribonucleotide binding; in biological processes, they were apoptosis, cell death, protein amino acid phosphorylation, and post-translational protein modification (Fig 6). Additionally, we found that the most enriched GO terms differed between hypo-methylated genes and hyper-methylated genes. Among the hyper-methylated genes, the most enriched GO terms were transferase activity, kinase activity, and phosphotransferase activity; among the hypo-methylated genes, they were protein binding, ATP binding, and adenyl ribonucleotide binding. These results suggest that different biological processes are activated during the day and night. The KEGG pathway analysis indicated that DMR-overlapped genes were mapped to carbohydrate metabolism processes, nucleotide metabolism processes, genetic information processing, environmental adaptation, and signal transduction pathways. Thus, although only a small amount of genes changed DNA methylation status between day and night, they were important in the diurnal response of poplar to the daily environment.

### Verification of certain DMRs and their effects on gene expression

The accuracy of MeDIP-seq was validated by bisulfite sequencing PCR (BSP) using the DNA samples for MeDIP-seq. Five genes that overlapped with DMRs in promoter regions were selected, including Potri.012G138800 (chalcone synthase-like protein), Potri.007G044000 (Glutamate-gated kainate-type ion channel receptor subunit GluR5), Potri.002G180100 (metal tolerance protein, PTRMTP2), Potri.001G189900 (histidine phosphotransfer protein) and Potri.003G099100 (Nbs-lrr resistance protein) (Table 1). The bisulfite sequencing data were consistent with the MeDIP-seq results (Table 4). For example, the MeDIP-seq data revealed that the methylation level in the 367-bp promoter region of Potri.012G138800 was hyper-methylated when 24:00 and 8:00 samples were compared. The bisulfite sequencing result showed that this region in the 24:00 samples contained 50 methylated cytocines (^m^C), whereas the number of ^m^C in 8:00 samples was 21, indicating that the 367-bp promoter region of Potri.012G138800 was hyper-methylated in the 24:00 samples, confirming the MeDIP-seq results.

**Table 4:** Verification of MeDIP-seq results by BS-PCR.

Overlapped Gene ID	MeDIP-seq			BS-PCR		
	8:00	24:00	8:00/24:00^a^	8:00	24:00	8:00/24:00^b^
Potri.012G138800	30	84	0.36	21	50	0.42
Potri.003G099100	42	13	3.23	41	33	1.24
Potri.002G180100	69	20	3.45	63	19	3.31
Potri.007G044000	68	25	2.72	74	22	3.36
Potri.001G189900	16	43	0.37	18	30	0.60

a: Methylation rate of MeDIP-seq was calculated as the amount of methylated reads in 8:00 divided by that in 24:00 samples.

b: Methylation rate of BS-PCR was calculated as the methylated cytocines divided by the total cytocines in the amplified PCR fragment.

**Fig 7:**
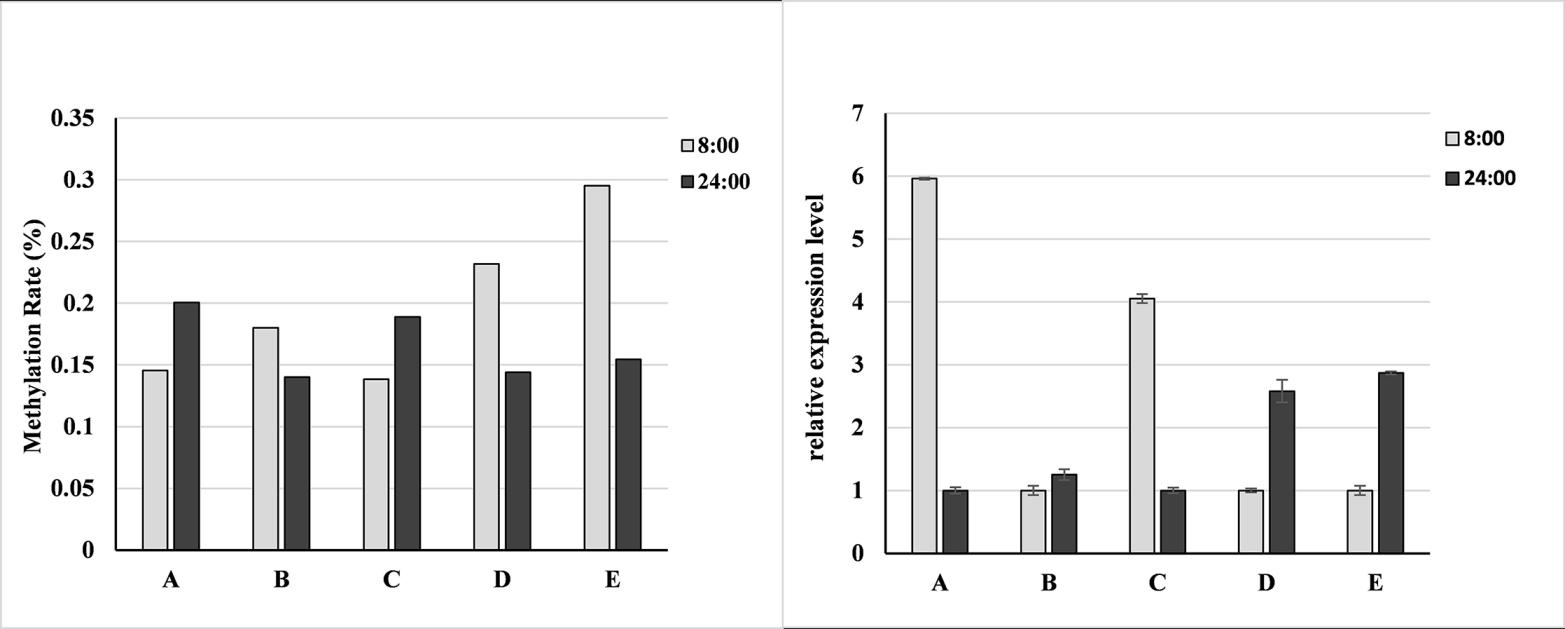
The methylation levels (left) and expression levels (right) of the five DMR-overlapped genes. A: POPTR_0001s19080 (Histidine phosphotransfer protein); B: POPTR_0003s09790 (Nbs-lrr resistance protein); C: POPTR_0012s13440 (Chalcone synthase-like protein); D: POPTR_0007s11010 (Glutamate-gated kainate-type ion channel receptor subunit GluR5); E: POPTR_0002s18080 (Metal tolerance protein, PTRMTP2). Methylation rate was calculated as ^m^C/total C of the amplified PCR fragment.

The expression levels of the five genes with DMRs in the promoter region were detected using qPCR. The expression levels of two hyper-methylated genes (*CHSL2* and the histidine phosphotransfer protein-coding gene) were significantly down-regulated in the 24:00 samples compared to the 8:00 samples. Conversely, the other three hypo-methylated genes exhibited the opposite expression patterns (Fig 7).

## Discussion

### DNA methylation was prevalent in the poplar genome and only a small portion of genes may be involved in diurnal regulation

The characterization of genome-wide methylation patterns in plant systems, particularly DNA methylation profile changes in response to various environmental conditions, has been a hot research area in recent years [32]. Many studies have focused on the altered DNA methylation induced by dramatic environmental changes, such as abiotic stress and chemical treatment [33]. However, there is limited information regarding methylation changes in response to minor environmental changes, such as altered light intensity and temperature. The present study is the first to systematically compare genome-wide methylation profiles in *P*. *nigra* mature leaves between day and night. Considering the average day length during the experimental period, we chose 8:00 and 24:00 as the two sample collection time points, to represent DNA methylation during the day and night of one natural day. Using the MeDIP-seq method, we obtained DNA methylation profiles for both time points, and numerous genes were found to be methylated in different genic regions, especially in the promoter regions, suggesting the potential role of DNA methylation in downstream gene regulation. These genes were shown to be involved in multiple biological processes, molecular functions, and cellular components, indicating the importance of the roles of DNA methylation in poplar cells.

Additionally, 2,120 DMRs were identified by comparing the 24:00 and 8:00 samples, with only 440 DMRs located in the genic region that can influence the expression of overlapped genes. Therefore, although several genes were found to be methylated in the *P*. *nigra* genome, only a tiny fraction of these changed methylation status during the day/night cycle. In hybrid aspen (*P*. *tremula* × *P*. *tremuloides*), approximately 1,695 were identified as diurnally regulated genes [34]. In the current study, only 137 DMR-overlapped genes were identified by comparing the 24:00 and 8:00 samples, indicating that only a small proportion of diurnally expressed genes might be regulated by DNA methylation in poplars.

Previous studies indicated that plants have evolved a range of gene regulatory mechanisms to adapt to various environmental changes during the day, such as changes in temperature and light intensity [35]. Due to their functional role in transcriptional regulation, together with our RT-qPCR results, the methylation changes in the small amount of genes detected in this study did appear to cause time-specific expression of these genes between day and night, but the exact functional role of DMRs in the day night cycle till needs further study. Therefore, although DNA methylation is not a main regulation pathway in plant diurnal rhythm, it might be a minor compensation to other regulation pathways in plant diurnal rhythm.

### The relationship between the methylation level and expression level was not linear

Previous studies have shown the relationship between methylation levels and corresponding gene expression in plants, with promoter methylation generally negatively correlated with gene expression, whereas gene body methylation positively correlated with gene expression [36,37]. In our study, BSP results of five genes with promoter regions overlapping with DMRs confirmed that the methylation levels in promoter regions repressed the expression of downstream genes. However, the effects of the DNA methylation levels of promoter regions on the expression of downstream genes were different. For example, the methylation level of promoter regions of the three genes (Nbs-lrr resistance protein coding gene, Potri.003G099100; metal tolerance protein coding gene, Potri.002G180100; and glutamate-gated kainate-type ion channel receptor subunit GluR5 coding gene, Potri.007G044000) decreased in 24:00 samples compared to those in the 8:00 samples. However, there was a significant difference in the expression levels of the three genes, with two genes (Potri.002G180100, Potri.007G044000) increasing by approximately 2.5–3.0 times, whereas one gene increased by only a small amount (Fig 5). Therefore, the DMRs in promoter regions have diverse effects on gene expression, and there is no direct linear relationship between the methylation level and expression level. This result may also explain those of several previous studies that showed only a small proportion of the DMR-overlapped genes exhibiting significantly different expression levels [38–40]. In most cases, the threshold to determine differentially expressed genes (DEGs) between samples was equal to or greater than a 1.5-fold change, and the expression differences in a large proportion of DMR-overlapped genes could not meet this criterion. We suggest that in such cases, the threshold for DEGs should be set lower because of the complicated relationship between DNA methylation and gene expression.

## References

[1] Henderson IR, Jacobsen, SE. Epigenetic inheritance in plants. Nature 2007; 447(7143): 418–424. doi: 10.1038/nature05917 17522675 10.1038/nature05917

[2] Law JA, Jacobsen SE. Establishing, maintaining and modifying DNA methylation patterns in plants and animals. Nature Reviews Genetics 2010; 11(3): 204–220. doi: 10.1038/nrg2719 20142834 10.1038/nrg2719 PMC3034103

[3] Chan SWL, Henderson IR, Jacobsen SE. Gardening the genome: DNA methylation in Arabidopsis thaliana. Nature Reviews Genetics 2005; 6(5): 351–360. doi: 10.1038/nrg1601 15861207 10.1038/nrg1601

[4] Huettel B, Kanno T, Daxinger L, Aufsatz W, Matzke AJ, Matzke M. Endogenous targets of RNA-directed DNA methylation and Pol IV in Arabidopsis. The EMBO journal 2006; 25(12): 2828–2836. doi: 10.1038/sj.emboj.7601150 16724114 10.1038/sj.emboj.7601150 PMC1500864

[5] Regulski M, Lu Z, Kendall J, Donoghue MT, Reinders J, Llaca V, et al The maize methylome influences mRNA splice sites and reveals widespread paramutation-like switches guided by small RNA. Genome research 2013; 23(10): 1651–1662. doi: 10.1101/gr.153510.112 23739895 10.1101/gr.153510.112 PMC3787262

[6] Tsuchiya T, Eulgem T. An alternative polyadenylation mechanism coopted to the Arabidopsis RPP7 gene through intronic retrotransposon domestication. Proceedings of the National Academy of Sciences 2013; 110(37): E3535–E3543. doi: 10.1073/pnas.1312545110 23940361 10.1073/pnas.1312545110 PMC3773791

[7] Dowen RH, Pelizzola M, Schmitz RJ, Lister R, Dowen JM, Nery JR, et al Widespread dynamic DNA methylation in response to biotic stress. Proceedings of the National Academy of Sciences 2012; 109(32): E2183–E2191. doi: 10.1073/pnas.1209329109 22733782 10.1073/pnas.1209329109 PMC3420206

[8] Mirouze M, Lieberman-Lazarovich M, Aversano R, Bucher E, Nicolet J, Reinders J, et al Loss of DNA methylation affects the recombination landscape in Arabidopsis. Proceedings of the National Academy of Sciences 2012; 109(15), 5880–5885. doi: 10.1073/pnas.1120841109 22451936 10.1073/pnas.1120841109 PMC3326504

[9] Melamed-Bessudo C, Levy AA. Deficiency in DNA methylation increases meiotic crossover rates in euchromatic but not in heterochromatic regions in Arabidopsis. Proceedings of the National Academy of Sciences 2012; 109 (16): E981–E988. doi: 10.1073/pnas.1120742109 22460791 10.1073/pnas.1120742109 PMC3341010

[10] Cubas P, Vincent C, Coen E. An epigenetic mutation responsible for natural variation in floral symmetry. Nature 1999; 401(6749): 157–161. doi: 10.1038/43657 10490023 10.1038/43657

[11] Soppe WJ, Jacobsen SE, Alonso-Blanco C, Jackson JP, Kakutani T, Koornneef M, et al The late flowering phenotype of fwa mutants is caused by gain-of-function epigenetic alleles of a homeodomain gene. Molecular cell 2000; 6(4): 791–802. 11090618 10.1016/s1097-2765(05)00090-0

[12] Stam M, Belele C, Dorweiler JE, Chandler VL. Differential chromatin structure within a tandem array 100 kb upstream of the maize b1 locus is associated with paramutation. Genes and development 2002; 16(15): 1906–1918. doi: 10.1101/gad.1006702 12154122 10.1101/gad.1006702 PMC186425

[13] Manning K, Tör M, Poole M, Hong Y, Thompson AJ, King GJ, et al A naturally occurring epigenetic mutation in a gene encoding an SBP-box transcription factor inhibits tomato fruit ripening. Nature genetics 2006; 38(8): 948–952. doi: 10.1038/ng1841 16832354 10.1038/ng1841

[14] Huh JH, Bauer MJ, Hsieh TF, Fischer RL. Cellular programming of plant gene imprinting. Cell 2008; 132(5): 735–744. doi: 10.1016/j.cell.2008.02.018 18329361 10.1016/j.cell.2008.02.018

[15] Martin A, Troadec C, Boualem A, Rajab M, Fernandez R, Morin H, et al A transposon-induced epigenetic change leads to sex determination in melon. Nature 2009; 461(7267): 1135–1138. doi: 10.1038/nature08498 19847267 10.1038/nature08498

[16] Tricker PJ, Gibbings JG, López CMR, Hadley P, and ilkinson MJ. Low relative humidity triggers RNA-directed de novo DNA methylation and suppression of genes controlling stomatal development. Journal of experimental botany 2012; 63(10): 3799–3813. doi: 10.1093/jxb/ers076 22442411 10.1093/jxb/ers076 PMC3733579

[17] Yamamuro C, Miki D, Zheng Z, Ma J, Wang J, Yang Z, et al Overproduction of stomatal lineage cells in Arabidopsis mutants defective in active DNA demethylation. Nature communications 2014; 5: 4062 doi: 10.1038/ncomms5062 24898766 10.1038/ncomms5062 PMC4097119

[18] Verhoeven KJ, Jansen JJ, Van Dijk PJ, Biere A. Stress-induced DNA methylation changes and their heritability in asexual dandelions. New Phytologist 2010; 185(4): 1108–1118. doi: 10.1111/j.1469-8137.2009.03121.x 20003072 10.1111/j.1469-8137.2009.03121.x

[19] Azzi A, Dallmann R, Casserly A, Rehrauer H, Patrignani A, Maier B, et al Circadian behavior is light-reprogrammed by plastic DNA methylation. Nature neuroscience 2014; 17(3): 377–382. doi: 10.1038/nn.3651 24531307 10.1038/nn.3651

[20] Hashida SN, Uchiyama T, Martin C, Kishima Y, Sano Y, Mikami T. The temperature-dependent change in methylation of the Antirrhinum transposon Tam3 is controlled by the activity of its transposase. The Plant Cell 2006; 18(1): 104–118. doi: 10.1105/tpc.105.037655 16326924 10.1105/tpc.105.037655 PMC1323487

[21] Naydenov M, Baev V, Apostolova E, Gospodinova N, Sablok G, Gozmanova M, et al High-temperature effect on genes engaged in DNA methylation and affected by DNA methylation in Arabidopsis. Plant Physiology and Biochemistry 2015; 87: 102–108. doi: 10.1016/j.plaphy.2014.12.022 25576840 10.1016/j.plaphy.2014.12.022

[22] Omidvar V, Fellner M. DNA methylation and transcriptomic changes in response to different lights and stresses in 7B-1 male-sterile tomato. PloS one 2015; 10(4): e0121864 doi: 10.1371/journal.pone.0121864 25849771 10.1371/journal.pone.0121864 PMC4388563

[23] Diao S, Wang Y, Ding C, Chang Y, Liang L, Gao Y, et al No consistent daily variation in DNA methylation detected in Populus nigra leaves by methylation-sensitive amplification polymorphism analysis. J. For. Res. 2017; 28(4): 653–660.

[24] Wang P, Xia H, Zhang Y, Zhao S, Zhao C, Hou L, et al Genome-wide high-resolution mapping of DNA methylation identifies epigenetic variation across embryo and endosperm in Maize (Zea may). BMC genomics 2015; 16(1): 21 doi: 10.1186/s12864-014-1204-7 25612809 10.1186/s12864-014-1204-7 PMC4316406

[25] Wang L, Feng Z, Wang X, Wang X, Zhang X. DEGseq: an R package for identifying differentially expressed genes from RNA-seq data. Bioinformatics 2010; 26(1): 136–138. doi: 10.1093/bioinformatics/btp612 19855105 10.1093/bioinformatics/btp612

[26] Livak KJ, Schmittgen TD. Analysis of relative gene expression data using real-time quantitative PCR and the 2− ΔΔCT method. Methods 2001; 25(4): 402–408. doi: 10.1006/meth.2001.1262 11846609 10.1006/meth.2001.1262

[27] Vining K, Pomraning KR, Wilhelm LJ, Ma C, Pellegrini M, Di Y, et al Methylome reorganization during in vitro dedifferentiation and regeneration of Populus trichocarpa. BMC plant biology 2013; 13(1): 92 doi: 10.1186/1471-2229-13-92 23799904 10.1186/1471-2229-13-92 PMC3728041

[28] Liu H, Ma L, Yang X, Zhang L, Zeng X, Xie S, et al Integrative analysis of DNA methylation, mRNAs, and small RNAs during maize embryo dedifferentiation. BMC Plant Biol. 2017; 17(1):105 doi: 10.1186/s12870-017-1055-x 28619030 10.1186/s12870-017-1055-x PMC5472921

[29] Dou L, Jia X, Wei H, Fan S, Wang H, Guo Y, et al Global analysis of DNA methylation in young (J1) and senescent (J2) Gossypium hirsutum L. cotyledons by MeDIP-Seq. PLoS One 2017; 12(7):e0179141 doi: 10.1371/journal.pone.0179141 28715427 10.1371/journal.pone.0179141 PMC5513416

[30] Yong-Villalobos L, González-Morales S. I, Wrobel K, Gutiérrez-Alanis D, Cervantes-Peréz SA, Hayano-Kanashiro C, et al Methylome analysis reveals an important role for epigenetic changes in the regulation of the Arabidopsis response to phosphate starvation. Proceedings of the National Academy of Sciences 2015; 112(52): E7293–E7302. doi: 10.1073/pnas.1522301112 26668375 10.1073/pnas.1522301112 PMC4702951

[31] Li SF, Zhang GJ, Zhang XJ, Yuan JH, Deng CL, Gu LF, et al DPTEdb, an integrative database of transposable elements in dioecious plants. Database (Oxford) 2016;1–10. doi: 10.1093/database/baw078 27173524 10.1093/database/baw078 PMC4865326

[32] Matzke MA, Mosher RA. RNA-directed DNA methylation: an epigenetic pathway of increasing complexity. Nature Reviews Genetics 2014; 15(6): 394–408. doi: 10.1038/nrg3683 24805120 10.1038/nrg3683

[33] Laird PW. Principles and challenges of genome-wide DNA methylation analysis. Nature Reviews Genetics 2010; 11(3): 191–203. doi: 10.1038/nrg2732 20125086 10.1038/nrg2732

[34] Hoffman DE, Jonsson P, Bylesjö MAX, Trygg J, Antti H, Eriksson ME, et al Changes in diurnal patterns within the Populus transcriptome and metabolome in response to photoperiod variation. Plant, cell and environment 2010; 33(8): 1298–1313. doi: 10.1111/j.1365-3040.2010.02148.x 20302601 10.1111/j.1365-3040.2010.02148.x

[35] Kinoshita T, Seki M. Epigenetic memory for stress response and adaptation in plants. Plant Cell Physiol. 2014; 55(11):1859–1863. doi: 10.1093/pcp/pcu125 25298421 10.1093/pcp/pcu125

[36] Zilberman D, Gehring M, Tran RK, Ballinger T, Henikoff S. Genome-wide analysis of Arabidopsis thaliana DNA methylation uncovers an interdependence between methylation and transcription. Nature genetics 2007; 39(1): 61–69. doi: 10.1038/ng1929 17128275 10.1038/ng1929

[37] Li X, Zhu J, Hu F, Ge S, Ye M, Xiang H, et al Single-base resolution maps of cultivated and wild rice methylomes and regulatory roles of DNA methylation in plant gene expression. BMC genomics 2012; 13: 300 doi: 10.1186/1471-2164-13-300 22747568 10.1186/1471-2164-13-300 PMC3447678

[38] Garg R, Chevala VN, Shankar R, Jain M. Divergent DNA methylation patterns associated with gene expression in rice cultivars with contrasting drought and salinity stress response. Sci. Rep. 2015; 5:14922 doi: 10.1038/srep14922 26449881 10.1038/srep14922 PMC4598828

[39] Secco D, Wang C, Shou H, Schultz MD, Chiarenza S, Nussaume L, et al Stress induced gene expression drives transient DNA methylation changes at adjacent repetitive elements. Elife 2015 7 21;4 doi: 10.7554/eLife.09343 26196146 10.7554/eLife.09343 PMC4534844

[40] Wang W, Qin Q, Sun F, Wang Y, Xu D, Li Z, Fu B. Genome-Wide Differences in DNA Methylation Changes in Two Contrasting Rice Genotypes in Response to Drought Conditions. Frontiers in Plant Science 2016; 7: 1675 doi: 10.3389/fpls.2016.01675 27877189 10.3389/fpls.2016.01675 PMC5099141

